# Estimating malaria transmission risk through surveillance of human–vector interactions in northern Ghana

**DOI:** 10.1186/s13071-023-05793-2

**Published:** 2023-06-19

**Authors:** Sylvester Coleman, Yemane Yihdego, Frank Gyamfi, Lena Kolyada, Jon Eric Tongren, Sixte Zigirumugabe, Dominic B. Dery, Kingsley Badu, Kwasi Obiri-Danso, Daniel Boakye, Daniel Szumlas, Jennifer S. Armistead, Samuel K. Dadzie

**Affiliations:** 1 U.S. President’s Malaria Initiative VectorLink Project, Accra, Ghana; 2 U.S. President’s Malaria Initiative, Malaria Branch, U.S. Centers for Disease Control and Prevention, Accra, Ghana; 3 U.S. President’s Malaria Initiative, U.S. Agency for International Development, Accra, Ghana; 4 Kwame Nkrumah University of Science and Technology, Kumasi, Ghana; 5 Noguchi Memorial Institute for Medical Research, University of Ghana, Legon, Accra, Ghana; 6 Armed Forces Pest Management Board, 172 Forney Road, Forest Glen Annex, Silver Spring, MD 20910 USA; 7 U.S. President’s Malaria Initiative, U.S. Agency for International Development, Washington, DC USA; 8 Department of Vector Biology, Liverpool School of Tropical Medicine, Pembroke Place, Liverpool, L3 5QA UK

**Keywords:** *Anopheles gambiae*, Malaria transmission risk, Human behavior observations, Bionomics, Insecticide treated nets(ITNs), Indoor residual spraying, Parity rate, EIR, Ghana

## Abstract

**Background:**

Vector bionomics are important aspects of vector-borne disease control programs. Mosquito-biting risks are affected by environmental, mosquito behavior and human factors, which are important for assessing exposure risk and intervention impacts. This study estimated malaria transmission risk based on vector–human interactions in northern Ghana, where indoor residual spraying (IRS) and insecticide-treated nets (ITNs) have been deployed.

**Methods:**

Indoor and outdoor human biting rates (HBRs) were measured using monthly human landing catches (HLCs) from June 2017 to April 2019. Mosquitoes collected were identified to species level, and *Anopheles gambiae* sensu lato (*An. gambiae* s.l.) samples were examined for parity and infectivity. The HBRs were adjusted using mosquito parity and human behavioral observations.

**Results:**

*Anopheles gambiae* was the main vector species in the IRS (81%) and control (83%) communities. Indoor and outdoor HBRs were similar in both the IRS intervention (10.6 vs. 11.3 bites per person per night [b/p/n]; z = −0.33, *P* = 0.745) and control communities (18.8 vs. 16.4 b/p/n; z = 1.57, *P* = 0.115). The mean proportion of parous *An. gambiae* s.l. was lower in IRS communities (44.6%) than in control communities (71.7%). After adjusting for human behavior observations and parity, the combined effect of IRS and ITN utilization (IRS: 37.8%; control: 57.3%) on reducing malaria transmission risk was 58% in IRS + ITN communities and 27% in control communities with ITNs alone (z = −4.07, *P* < 0.001). However, this also revealed that about 41% and 31% of outdoor adjusted bites in IRS and control communities respectively, occurred before bed time (10:00 pm). The mean directly measured annual entomologic inoculation rates (EIRs) during the study were 6.1 infective bites per person per year (ib/p/yr) for IRS communities and 16.3 ib/p/yr for control communities. After considering vector survival and observed human behavior, the estimated EIR for IRS communities was 1.8 ib/p/yr, which represents about a 70% overestimation of risk compared to the directly measured EIR; for control communities, it was 13.6 ib/p/yr (16% overestimation).

**Conclusion:**

Indoor residual spraying significantly impacted entomological indicators of malaria transmission. The results of this study indicate that vector bionomics alone do not provide an accurate assessment of malaria transmission exposure risk. By accounting for human behavior parameters, we found that high coverage of ITNs alone had less impact on malaria transmission indices than combining ITNs with IRS, likely due to observed low net use. Reinforcing effective communication for behavioral change in net use and IRS could further reduce malaria transmission.

**Graphical Abstract:**

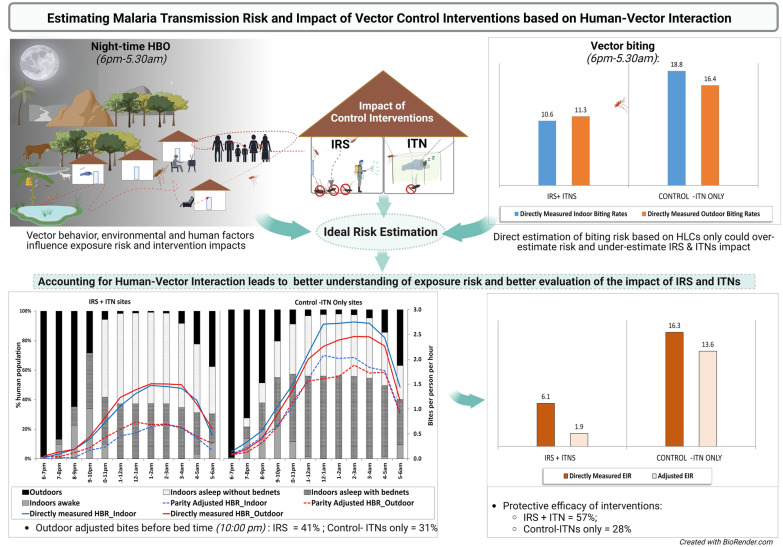

**Supplementary Information:**

The online version contains supplementary material available at 10.1186/s13071-023-05793-2.

## Background

Effective malaria vector control relies mainly on two core insecticide-based interventions: deployment of insecticide-treated nets (ITNs) and indoor residual spraying (IRS) of insecticides [1]. Increased global investments for malaria control led to the increase in coverage of ITNs and IRS across sub-Saharan Africa between 2000 and 2015 [2]. This increase is reported to have contributed to a 37% decline in overall malaria incidence as well as a 60% reduction in malaria mortality rates globally during the same period (2000–2015) [3]. Recent estimates however indicate that progress in malaria control, particularly in high-burden countries, has slowed and then remained stagnant in the last 4 years [4]. This has been attributed to a myriad of interrelated challenges described as either biological, socioeconomic or political [5–7]. Biological challenges, such as behavioral adaptations of vectors, resistance to insecticides and non-adoption of desired malaria prevention methods, remain the most important challenges since they have direct and indirect consequences that threaten the recent gains in malaria control [3, 8–10].

Some malaria vector species that have undermined insecticidal controls such as IRS and ITN programs exhibit one or more of the following behavioral traits: feeding and resting outdoors, early exit behavior and/or opportunistic feeding upon animals rather than humans [11]. Such feeding and resting behaviors of malaria vectors reduce protection derived from sleeping under ITNs during peak biting times or resting in houses treated with IRS [11], and can sustain malaria transmission referred to as “residual” transmission. Human–vector interaction also plays a key role in malaria transmission [12–16]. Individuals who spend significant time outdoors participating in night-time activities or sleeping without a bed net could be more at risk of acquiring malaria.

In northern Ghana, *Anopheles gambiae* sensu lato (*An. gambiae* s.l.) is the predominant vector; it has an equal tendency (i.e. 50%) to feed inside and outside at any given time when there are available hosts [17, 18], and outdoor human behaviors may be significant at peak biting times of infective mosquitoes [19]. Heterogeneity of both human and vector behavior may lead to an underestimation of true malaria transmission risk in a population, and ultimately considerable exposure of people assumed to be protected by ITNs or IRS against infective mosquito bites [20, 21].

This study was conducted to document how vector feeding behavior overlaps with human behavior and to assess its impact on malaria transmission dynamics in the northern savannah zone of Ghana where IRS and ITNs have been co-deployed for the last decade. Since 2018, the IRS intervention site has been exempt from ITN mass and school-based distribution but nets are available at health facilities through continuous distribution. We compared the human–vector interactions and estimated the transmission risk indices in two districts of northern Ghana with different IRS and ITN histories, by simultaneously monitoring hourly vector and human behavior indoors and outdoors. Findings from human–vector behavior studies can be used to determine whether outdoor biting is occurring because humans are outdoors, and to determine the protective impact of vector control tools and whether alternative tools are needed if ITNs and IRS are not protecting humans from biting/transmission.

## Methods

### Study sites

**Fig. 1:**
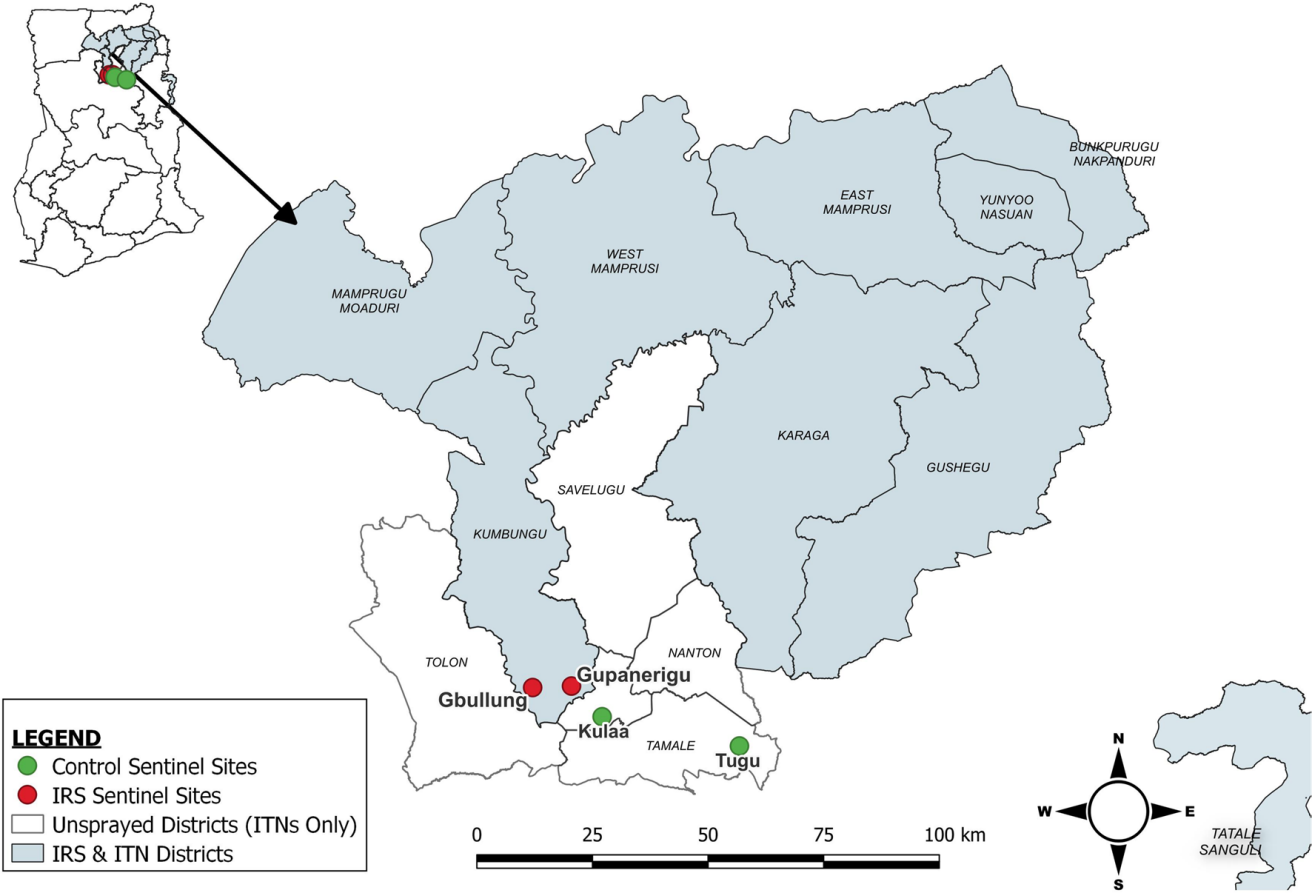
The study was conducted in northern Ghana in IRS (light-blue shading) and unsprayed (no shading) districts. Human and vector behavior observations were collected from two IRS communities (red symbols) in Kumbungu district and two control communities (green symbols) in Tamale Metropolitan District. IRS, Indoor residual spraying; ITN, insecticide-treated net

The study was conducted in four communities (sites) in northern Ghana: two IRS communities in Kumbungu district (Gbullung and Gupanarigu), where U.S. President’s Malaria Initiative (PMI) supported IRS from 2008 to 2012 and again from 2015 to the present, and two control communities (Kulaa and Tugu) located on the outskirts of Tamale metropolis, which have no history of IRS (Fig. 1; Additional file 1: Table S1). The communities are rural and have similar vector ecology, community demographics and weather/climate/vegetation pattern. They also have similar housing structures, with most houses made of thatched roofs and mud walls; few houses with cement/concrete wall and zinc roofs exist in these communities, and these are mostly in Tugu. Farming is the main livelihood occupation of inhabitants.

### Vector control interventions in the study area

In both 2017 and 2018 both Gbullung and Gupanarigu were sprayed with an organophosphate insecticide (pirimiphos methyl CS formulation at a rate of 1 g/m^2^) with a residual life of 7 months, covering the entire malaria transmission season [22]. The IRS campaigns in both years were timed to coincide with the first or second month of the rainy season (March/April) depending on the year [23].

All four communities received ITNs delivered through mass distribution campaigns in 2010, 2012 and 2016. ITN coverage in intervening years has been maintained through continuous distributions in child welfare clinics and antenatal clinics since 2013. Whereas the control communities benefitted from a mass ITN distribution in 2018, the IRS communities were exempted. Available data show ITN usage among pregnant women and children under 5 years of age in the IRS communities to be 59.4% and 58.4%, respectively [24]. Before the current study commenced in June 2017, the percentage of the population in the northern region with access to ITNs was 77%, but actual usage was estimated to be 51% [25]. The control communities were expected to have similar ITN usage of 51% before the study started.

### Study period

The study was conducted longitudinally over a period of 22 months, from June 2017 to April 2019.

Monthly mosquito collections were conducted over two transmission seasons, June 2017 to April 2018 and June 2018 to April 2019, capturing two distinct seasonal periods: wet/rainy (June–October) and dry (December–April), with 1-month breaks in November and May of each year. A total of 10 houses per community were randomly selected for monthly human landing catches (HLCs). Of the 10 houses per community, six were selected for human behavior observations during HLCs. Direct observations of night-time activities were conducted alongside HLCs monthly.

### Meteorological data collection

Mean daily rainfall data from the study area was obtained from weather stations of the Ghana Meteorological Services Department in Tamale and from the Savannah Agricultural Research Institute in Nyankpala. The amount of rainfall recorded 2 weeks prior to the data collections was compared with human biting rates (HBRs) to assess the correlation between rainfall and biting rates of the predominant vector, *An. gambiae* s.l. Mean rainfalls of 79.5 mm and 74.6 mm were recorded for the IRS and control communities, respectively, in year 1 of the study. In year 2, however, the rainfall increased to 87.5 mm in IRS communities (10% increase) and to 99.3 mm (33% increase) in the control communities.

### Mosquito collection

Monthly mosquito collections were conducted simultaneously across all study sites using HLCs. HLCs were performed for a total of 4 nights (collecting mosquitoes hourly from 6:00 pm to 6:00 am) in each community per month. Two mosquito collectors worked in two teams of four persons (2 indoors and 2 outdoors) per house simultaneously each night (3 houses on days 1 and 3, and from 2 houses on days 2 and 4). A total of 40 man-nights (20 man-nights indoor and 20 man-nights outdoor) were used for mosquito collection per community each month. The same houses were used for data collection every month during the study. Indoor and outdoor mosquito collectors switched positions every hour and were allowed to take 10-min breaks between shifts. For the indoor collections, the collectors worked in rooms without mosquito nets and stayed in the room alone during the time of collections.

### Mosquito species identification

*Anopheles* mosquitoes were preliminarily identified morphologically to species using the keys of Gillies and Coetzee [26]. A sub-sample (about 5%) of total *An. gambiae* s.l. collected by HLCs was randomly selected and further identified to species by PCR analyses of ribosomal DNA [27]. Specimens identified as *Anopheles gambiae* sensu stricto (*An gambiae* s.s.) were further distinguished as either *Anopheles coluzzii* or *An. gambiae* following the restriction fragment length polymorphism–PCR procedure described by Fanello et al. [28].

### Sporozoite detection

The heads and thoraces of about 15% (average: 50 per site per month) of *An. gambiae* s.l. collected from HLCs monthly were sorted and tested for the presence of *Plasmodium falciparum* sporozoite circum-sporozoite antigens using an enzyme-linked immunosorbent assay as described by Wirtz et al. [29] to determine infective (sporozoite) parasites in the local vectors collected [29].

### Parity determination

About one-third (average: 50–60 mosquitoes per site per month) of unfed *An. gambiae* s.l. mosquitoes from HLCs from each site every month were dissected to assess parity by observing the degree of coiling in the ovarian tracheoles [30].

### Human behavior observations

A total of 24 houses (12 houses in the IRS communities and 12 houses in the control communities) were used for the direct observations each month. Night-time indoor and outdoor activities of 394 residents (156 from the control communities and 238 from intervention communities) were directly observed. These residents comprised 51.3% females and 48.7% males. Direct human behavior observations were also conducted in the same houses where the HLCs were performed to estimate the risk/exposure to residents.

One note taker was stationed per house to observe an average of 15 inhabitants. Trained note takers observed the activities of all household residents without interfering with their routine activities from 6:00 pm until 6:00 am. The direct observations included the time that people were indoors or outdoors, the types and duration of activities occurring outdoors during the night, when and where people slept and whether they were under a net, regardless of whether in/out. All observations were recorded for each hour on a household observation form (Additional file 2: Form S1). An inventory of bed nets per household was recorded during the listing of household members in the first month of observation. Utilization of the bed nets by household members over the study period was noted and recorded during each night of observation.

### Indicators and statistical analysis

The following entomological indicators were estimated for the malaria vectors collected:

- HBR refers to the total number of mosquitoes collected by HLC/number of mosquito collectors/number of collection nights (reported as bites per person per night [b/p/n]) [31]. HBRs were also calculated as indoor versus outdoor, both hourly and monthly.
  $${\text{Endophagic/exophagic index}} = \frac{{\text{Number of mosquitoes species collected (either indoors or outdoors)}}}{{\text{Total number of mosquitoes collected indoors and outdoors}}}$$.
- Parity rates were estimated as:
  $$\frac{{\text{Number of parous female mosquitoes}}}{{\text{Total number of female mosquitoes dissected}}}$$.
- Vector survival. The probability of a mosquito surviving 1 day after a blood meal and expectation of life for a mosquito are known to affect the likelihood of being bitten by an infective mosquito. By assuming a gonotrophic cycle of 2 days, the probability of vectors surviving through 1 day (*p*) is estimated as:

- $$p = \sqrt {\text{proportion parous}}$$
- The expectation of life is also estimated as: $\frac{1}{ - \ln p}$.

- Sporozoite rate refers to the proportion of mosquitoes that test positive for circumsporozoite proteins of *P. falciparum* per period per site.
- Entomological inoculation rate (EIR) is the number of infective bites received by an individual in an area within a period (either as months or annual EIRs). This was estimated for a specific site or district as follows:

- Monthly EIR = monthly HBRs × monthly sporozoite rates.
- Annual EIR (for March–December only) = sum of monthly EIRs.

- Estimating human behavior observation (HBO) and parity adjusted indicators:

- Directly measured HBRs indicate exposure risk but overlook the effects interventions like IRS and ITNs, which work by reducing mosquito longevity. Directly measured, HBRs estimates, which include both parous and nulliparous mosquitoes—as is the current practice—may overestimate risk and underestimate intervention effects. Since older vectors are more likely to transmit malaria, parity- and HBO-adjusted rates provide a more accurate representation of the exposure communities face.
- HBO-adjusted HBRs were estimated according to the method of Martin et al. [32], using the directly observed hourly mosquito biting rates and night-time HBOs—whether household residents were indoors or outdoors and the time they went to sleep. The resulting biting rates were then multiplied by the hourly parity data for each site to obtain the parity- and HBO-adjusted biting rates.

Data collected were analyzed using STATA version 15 software (2017; StataCorp, College Station, TX, USA). Linear hierarchical regression was used to calculate average differences in HBR (both directly measured and adjusted) between sprayed and control communities. In the linear hierarchical regression, the type of treatment (sprayed or unsprayed) was included as the main outcome of interest, with month of observation as a fixed effect and community and household as random effects. The Z-test for difference in proportions was also used to compare the differences in mean parity and sporozoite rates between *An. gambiae* s.l. populations from IRS and control communities. Only female *An. gambiae* s.l. mosquitoes were considered in the analyses.

## Results

### Mosquito species composition

**Fig. 2:**
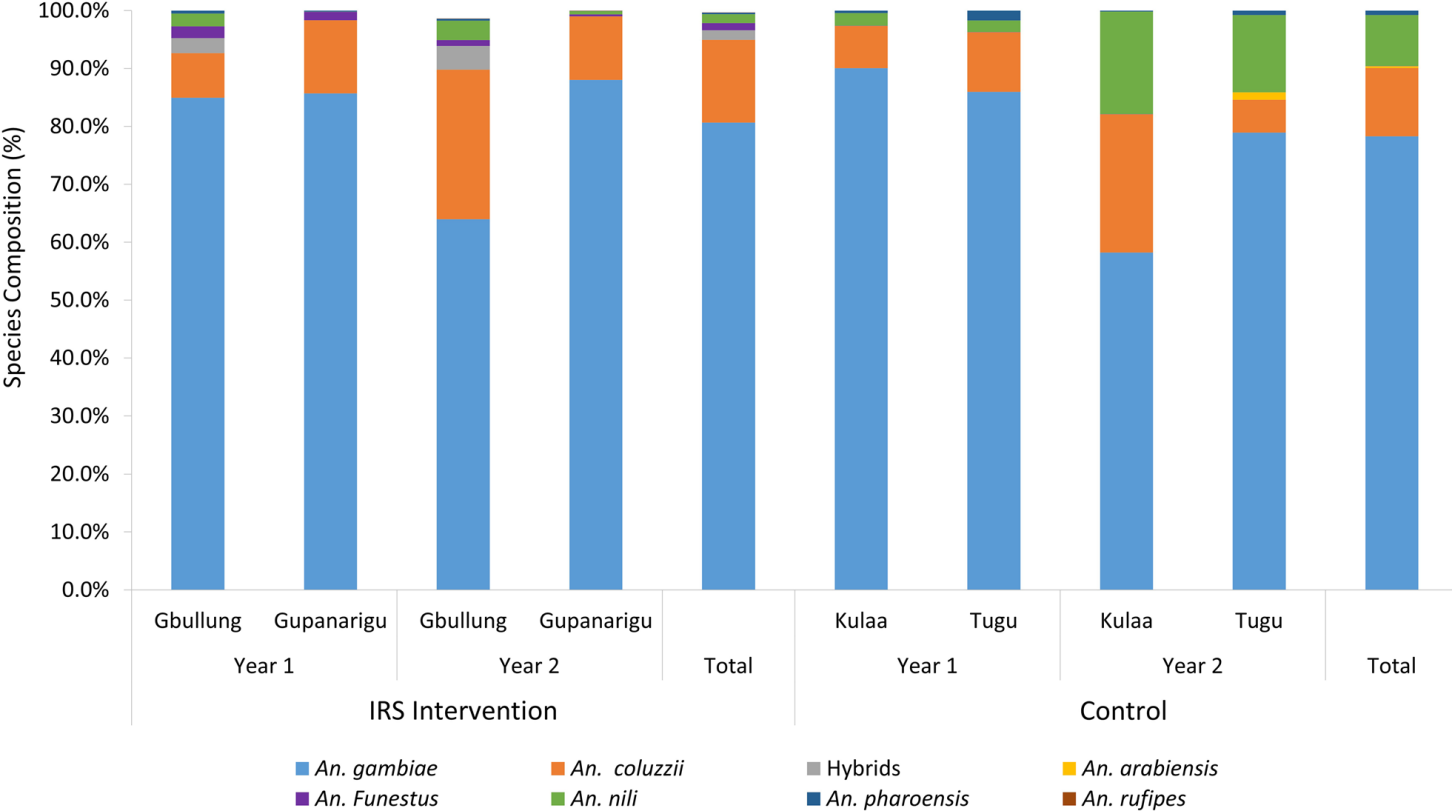
*Anopheles* species composition in IRS and control communities. *Anopheles gambiae* sensu lato (blue) was the predominant *Anopheles* species collected in the IRS and control communities in both years of the study. A sub-sample of *An. gambiae* sensu lato collected throughout the study period across all study sites primarily consisted of *An. gambiae* (blue), with equal proportions of *An. coluzzii* (orange) in both IRS and control communities and *An. arabiensis* (yellow) only detected in the control communities. IRS, Indoor residual spraying

A total of 48,735 *Anopheles* mosquitoes, comprising *An. gambiae* s.l. (93.6%), *An. nili* (5.40%), *An. funestus* sensu lato (*An. funestus* s.l.; 0.43%,), *An. pharoensis* (0.56%) and *An. rufipes* (0.01%), were collected over the study period using HLCs. The proportions of *An. gambiae* s.l. collected in years 1 and 2 in the IRS communities were similar (z = 1.48, *P* = 0.140), but in the control communities a lower proportion of *An. gambiae* s.l. was collected in year 2 compared to year 1 (z = −44.13, *P* < 0.001) while the proportion of *An. nili* increased from 2.1% in year 1 to 16% in year 2 (z = 5.71, *P* < 0.001). Most *An. funestus* s.l. (a secondary vector) collected were from IRS intervention communities (Fig. 2; Additional file 1: Table S2). None of the other *Anopheles* species (*An. nili*, *An. pharoensis* and *An. rufipes*) have been implicated in malaria transmission in the study area.

The subset of *An. gambiae* s.l. that were further identified by molecular analyses revealed that *An. gambiae* was the majority species in both the IRS (81%, *n* = 162/200) and control (83%, *n* = 280/338) communities. Similar proportions of *An. coluzzii* (16%) were identified in both study areas. Hybrids of *An. coluzzii* and *An. gambiae* were only collected from the IRS communities while all of the *An. arabiensis* collected were from the control communities.

### Vector survival estimation

The proportion of parous *An. gambiae* s.l. females was significantly higher (z = −20.35, *P* < 0.001) in the control than in the IRS communities (Table 1; Additional file 1: Table S3; Additional file: Figure S1).

**Table 1:** Estimated probability of surviving 1 day and expectation of life for *Anopheles gambiae* sensu lato collected from indoor residual spraying and control communities

Parameter	IRS communities	Control communities
Mean parity (%)	44.6^a^ (*n* = 2334)^b^	71.7^a^ (*n* = 3226)^b^
Probability of vectors surviving through 1 day (*p*) assuming 2 days of gonotrophic cycle (%)	66.9	84.7
Probability of vectors surviving through the sporogonic cycle assuming 10 days (*p*^10^) (%)	0.03	3.6
Average expectation of life	1.2 days	3 days

Results for Z-test of proportion for parity indoor residual spraying (IRS) versus control. Pooled sample proportion: 0.6035, *P*-value: < 0.001, Z-test statistic: − 20.35

^a^Differences in mean parity significant at 0.05 significance level

^b^*n* refers to the number of* Anopheles gambiae* sensu lato dissected

Based on parity rates, the probability of a vector in the study area surviving a 10-day sporogonic cycle for *P. falciparum* was estimated to be 0.03% in the IRS communities compared to 3.6% in the control communities. Vectors in IRS communities had 67% chance of surviving through 1 day as compared to an 85% chance of surviving 1 day in the control site. Thus, vectors are expected to have a shorter life span in the presence of IRS (IRS communities) compared to the control unsprayed communities (Table 1).

### Vector biting behavior

The cumulative average daily HBR was 9.8 (± 1.3) b/p/n for IRS communities and 22.2 (± 2.4) b/p/n for the unsprayed control communities in year 1. Controlling for fixed effects for month, random effects for community, household and place of collection (indoor/outdoor), mean HBR was significantly higher for the control communities than for the IRS communities (z = −5.44, *P* < 0.001) in year 1. In year 2, there was no statistically significant difference (z = −0.33, *P* = 0.740) between the HBR in the IRS (12.1 b/p/n) and control (12.9 b/p/n) communities. HBR in the IRS communities increased from 9.8 to 12.1 b/p/n in year 2 (z = −1.32, *P* = 0.187), whereas the HBR in the unsprayed areas decreased significantly from 22.2 to about 12.9 b/p/n (z = −4.83, *P* < 0.001).

**Fig. 3:**
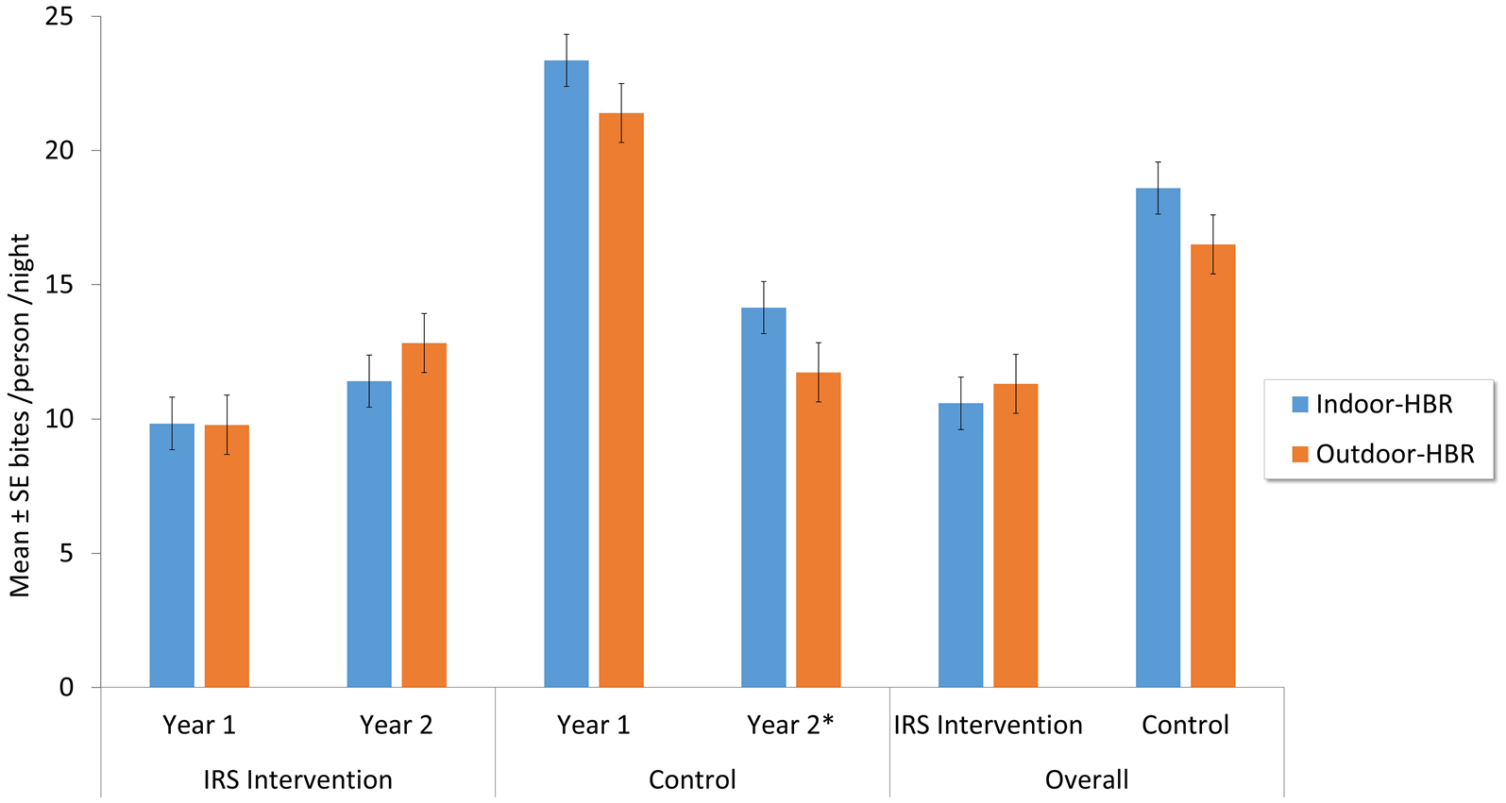
*Anopheles gambiae* sensu lato HBRs in the IRS and control communities. Mean indoor (blue) and outdoor (orange) HBRs (mean ± SE bites per person per night) of *An. gambiae* sensu lato as estimated from human landing catches varied, with significantly higher indoor biting in control communities in both years and marginally higher outdoor biting in IRS communities in year 2. HBRs, Human biting rates; IRS, Indoor residual spraying

In year 1, the indoor/outdoor proportion of *An. gambiae* s.l. was similar in the IRS communities (z= –0.02, *P* = 0.980), but in year 2 *An. gambiae* s.l. in the IRS communities showed slightly more exophagic tendencies (z = –0.87, *P* = 0.386). The opposite trend was observed in the control communities in both years. The differences in the proportions of *An. gambiae* feeding indoors versus outdoors were statistically significant in year 2 in the control communities (z = –3.19, *P* = 0.001) (Fig. 3). The mean bites per person per month (b/p/m) was 334 b/p/m for the IRS intervention communities and 538 b/p/m for the control communities (Additional file 1: Figure S2). HBRs of *An. gambiae* s.l. increased with the onset of the rains and declined markedly during the dry season.

### Human behavior observations

**Fig. 4:**
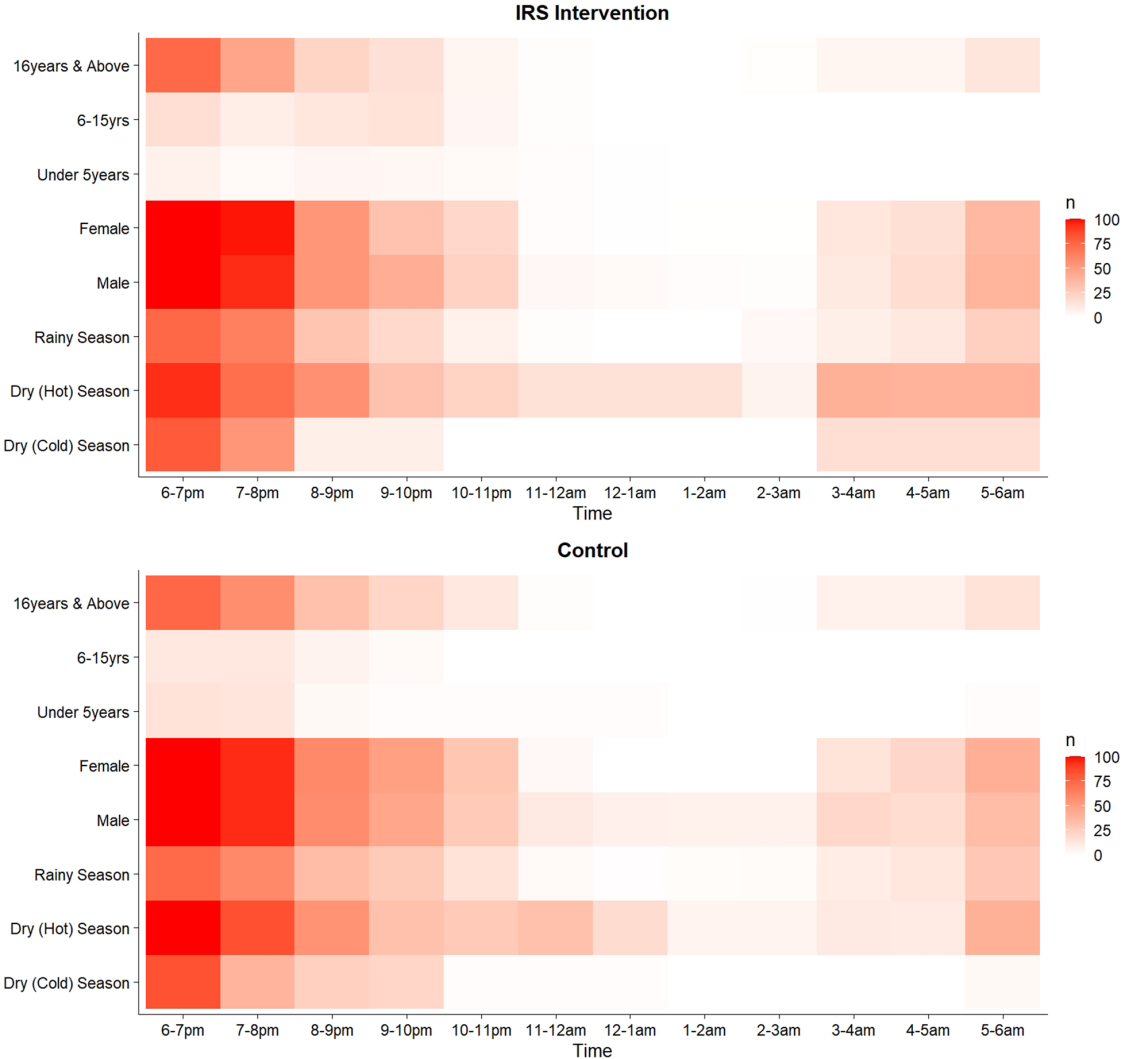
Proportion of residents observed engaging in outdoor night-time activities that increase the risk of malaria transmission, disaggregated by age, sex and season, in the IRS Intervention (top) and control (down)  communities. Heat map color intensity corresponds to outdoor activities with increased malaria risk, presented in ranges of 0, 0–25, 25–50, 50–75 and 75–100%. The proportions represent the percentage of the total household residents in each risk stratum on the *Y*-axis observed to be outdoors during a given hour of the night. Risky activities include sleeping outdoors without a bed net, watching videos, eating outdoors, storytelling and playing oware (a local board game). IRS, Indoor residual spraying

**Fig. 5:**
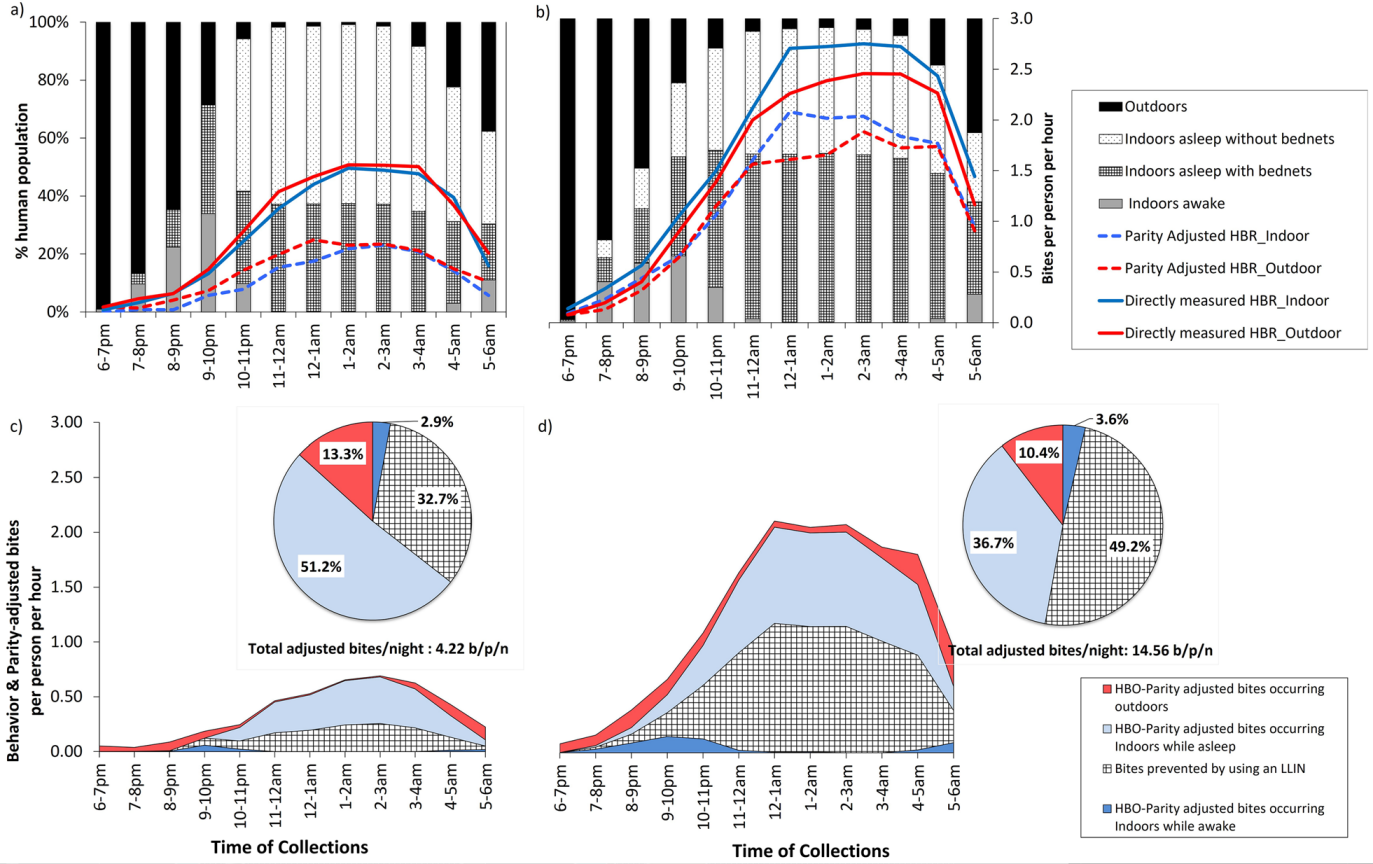
**a**,** b** Directly measured biting rate compared to parity-adjusted biting rates of *Anopheles gambiae* sensu lato and human location in the indoor residual spraying (IRS) communities (**a**) and in the control communities (**b**).** c**,** d** Human behavior-adjusted exposure to *An. gambiae * sensu lato bites during the rainy months in IRS communities (**c**) and in control communities (**d**). Mosquito biting data were collected using human landing catches (HLCs) conducted from 6:00 pm to 6:00 am in northern Ghana from June 2017 to April 2019, and human behavioral data were collected in parallel by direct observation during HLCs. The proportion of residents that were outdoors (black bars), indoors but awake (gray bar) or indoors and asleep with insecticide-treated nets (ITNs; hashed bars) and without ITNs (dotted bars) is shown. These data are overlaid with indoor (blue, dashed lines) and outdoor (red, dashed lines) biting rates of *An. gambiae* sensu lato after adjusting for mosquito parity. Exposure is shown as average hourly biting rates (bites per person per hour) as determined from the numbers of parous mosquitoes collected during HLCs conducted from 6:00 pm to 6:00 am during the rainy season, from June 2017 to April 2019. HBRs occurring outdoors (red), indoors while asleep (light blue) and indoors while awake (dark blue), as well as bites prevented by sleeping under an ITN (hashed) are shown; pie chart insets show proportion of total bites for each category. HBO, Human behavior observations; HBR, human biting rate; LLIN, long-lasting insecticidal net

Most (79.5%) household residents were observed to go to bed between 9:00 pm and 11:00 pm and woke up mostly between 4:00 am and 500 a.m. in preparation for dawn prayers (Figs. 4, 5). Residents spent more time outdoors at night during the dry, hot season (from February to mid-April) when the daily average temperature often exceeded 35.6 °C [33, 34] than during the rainy season or the dry, cold season (Fig. 5). During the cold harmattan season (December through early February) the most preferred time to retire to bed (inside) was between 8:00 pm and 9:00 pm.

The main activities that kept respondents outdoors (after 11:00 pm) were recreational (32%) and social (62%) in both seasons, suggesting an overlap between vector and human outdoor activity. Recreational activities included watching videos, storytelling and playing oware (a local board game). Examples of social activities that residents were engaged in included attending funerals, community meetings and festivals, or preparing for child naming ceremonies and weddings. In general, males in most communities stayed outdoors later than females.

### ITN ownership and use

Insecticide-treated net ownership across all study sites was approximately 80%, except for Gupanarigu, one of the IRS communities, where ITN ownership was just below 70%. ITN use varied by site. ITN usage was significantly lower in the IRS communities than in the control communities. Indoor net use rate was significantly lower in the IRS (37.8%) than in the control communities (57.3%) (z = −3.798, *P* < 0.001) (Table 2).

**Table 2:** Observed bed net usage in indoor residual spraying and control communities, June 2017–April 2019

Study site	Sample size (*n*)	Net ownership (%)	Indoor net use (%)	Outdoor net use (%)
*IRS communities (total)*	238	78.2	37.8	4.3
Gbullung	150	84.3	31.5	3.7
Gupanarigu	88	69.7	48.4	5.0
*Control (non-IRS)communities (total)*	156	81.4	57.3	21.2
Kulaa	100	81.6	53.6	13.2
Tugu	56	80.9	63.8	32.1

Results for Z-test comparing the net ownership and usage amongst observed residents in IRS versus control				
Parameter	Pooled sample proportion	Standard Error	Z test Statistic	*P*-value
Net ownership	0.794	0.042	− 0.783	0.434
Indoor net use	0.455	0.051	− 3.798	< 0.001
Outdoor net use	0.109	0.032	− 5.278	< 0.001

*IRS* Indoor residual spraying

### Adjusted estimates of malaria transmission risk

Comparable biting trends were observed for *An. gambiae* s.l. in both the IRS and control communities, with the most intense biting activity occurring between 1:00 am and 3:00 am in both groups (Fig 5. Additional file 1: Figure S3). The mean biting rates of *An. gambiae* s.l. for year 1 and 2 were 0.82 bites per hour (b/p/hr) indoors and 0.88 b/p/hr outdoors in the IRS communities.

#### Parity-adjusted biting rates

After adjusting for parity to estimate potential exposure to infectious mosquitoes, the average hourly indoor and outdoor biting rates for *An. gambiae* s.l. decreased to 0.33 b/p/hr (z = −3.75,* P* < 0.001) and 0.42 b/p/hr  (z = −3.65, *P * < 0.001), respectively, which were statistically significant differences. In control communities without IRS, a small decrease was observed when adjusting for parity. The average directly measured indoor biting rate decreased from 1.71 to 1.23 b/p/hr (z = −2.47, *P* = 0.015), while the outdoor biting rate declined from 1.50 to 1.12 b/p/hr (z = −1.95, *P* = 0.057).

#### Human behavior and parity-adjusted biting rates

Four main behavioral patterns were identified during the direct observations throughout the night, including people staying inside their rooms or outside in the open courtyard of the HLC houses, their sleeping schedules and the use of ITNs. These patterns form the HBO, which we used to adjust the biting rates and estimate the number of mosquito bites experienced by different groups.

By adjusting for parity and HBO, the total mean adjusted bites (for year 1 and 2) (i.e. the risk of being bitten by an adult mosquito capable of transmitting malaria per night) decreased by about 61.4% in the IRS intervention communities (from 10.9 to 4.2 b/p/n), while only a 17% decrease was recorded in the control communities (from 17.6 to 14.5 b/p/n) (Table 3). This translates into 131 bites/person/month (b/p/m) in the IRS communities and about 451 b/p/m in the control communities (Table 3). About 41% and 31% of the HBO- and parity-adjusted outdoor bites occurred before 10:00 pm in the IRS and control communities, respectively. The combined efficacy of IRS and ITNs in reducing exposure to potential infective bites based on HBO- and parity-adjusted rate was estimated to be 58% in IRS communities compared to 27% efficacy of ITNs alone in the control communities (Table 3). In the linear hierarchical regression analysis of HBO- and parity-adjusted rates, a statistically significant negative association with IRS + ITNs was observed. The treatment coefficient was −9.73 (z = −4.07, P < 0.001) indicating a significant decrease in HBO- and parity-adjusted rates, after adjusting for potential cluster effects within the communities. By accounting for HBO and parity, we found that estimated risk of exposure to potentially infective bites based on directly measured biting rates was overestimated by about 61% in the IRS intervention communities and by 17% in the control sites. About 51% of the HBO- and parity-adjusted bites (2.2 b/p/n of 4.2 b/p/n) in the IRS communities occurred indoors while people were asleep (Fig. 5).

**Table 3:** Directly measured and adjusted biting rates for *Anopheles gambiae* sensu lato in indoor residual spraying and control communities during the rainy season

Description	IRS communities	Control communities
*Directly measured biting rate of* *An. gambiae sensu lato*		
Mean biting rates per night (b/p/n): (đɱ_*hbr*_)	10.9	17.6
Mean biting rates per month (b/p/m)	337.9	545.6
Indoor/outdoor bite ratio	0.94	1.14
Proportion of bites occurring before 10:00 pm (indoors/outdoors)	7.2/7.9%	10.2/8.8%
*HBO-adjusted biting rate of* *An. gambiae sensu lato*		
Total HBO-adjusted bites (b/p/n): (ћβ_t_)	10.0	20.0
*HBO- and parity-adjusted biting rate of An. gambiae sensu lato*		
Total HBO and parity-adjusted per night (b/p/n): (ћβ_p_)	4.2*	14.6
Total HBO and parity-adjusted per month (b/p/m)	130.9	451.5
Proportion of bites occurring before 10 pm (in/out)	2.9/40.7%	8.4/30.6%
Proportion of bites prevented based on HBO and parity-adjusted biting rate for ITN users: [(ћβ_t_) − (ћβ_p_)]/(ћβ_t_)	58.1%*	27.0%
Degree of over-estimation (difference between HBO- and parity-adjusted and directly measured biting rates: [(đɱ_*hbr*_)-(ћβp) ]/(đɱ_*hbr*_)	61.5%	17.0%

Human behavior observations (HBO)-adjusted bites = proportion of inhabitants involved in the four core activities × directly measured biting rates

HBO- and parity-adjusted bites = HBO-adjusted bites × mean parity rates

Proportion of people using insecticide-treated nets: IRS communities = 37.8%; control communities = 57.3%

*Significant difference between communities at* P* < 0.05

*b/p/n* Bites per person per night,* ITN* insecticide-treated net

#### Sporozoite infections and entomological inoculation rates

Of 7067 *An. gambiae* s.l. collected by HLC and tested for the presence of sporozoites, 0.49% from the control communities and 0.26% from the IRS communities were positive for *P. falciparum* sporozoites.

*Anopheles gambiae* accounted for most of the infections in IRS and control communities, with five infections in the IRS communities and 20 infections in the control communities. Only one *An. coluzzii* was positive for sporozoites in the IRS communities, while three *An. coluzzii* were positive in the control communities. Sporozoite-positive mosquitoes were detected in samples collected between 10:00 pm and 4:00 am (Additional file 1: Figure S2). All infective mosquitoes collected indoors occurred between 10:00 pm and 1:00 am in both control and IRS communities, while infective mosquitoes collected outdoors were recorded between 10:00 pm and 4:00 am. There was no significant difference between indoor and outdoor sporozoite rates in the IRS intervention communities (z = 0.82, *P* = 0.414) or the control communities (z = − 0.15, *P* = 0.884). Fewer mosquitoes were analyzed for the IRS intervention site in the dry season because of fewer mosquitoes (77 out of 17,500 *An. gambiae* s.l. collected), hence these were excluded from the comparison in Additional file 1: Figure S3.

The mean annual EIR (calculated from the sum of monthly EIRs; Additional file 1: Figure S2) for the control communities was 16.3 infective bites per person per year (ib/p/yr), while the mean EIR for the IRS communities was about 6.1 ib/p/yr. After considering mosquito survival rates and human behavior patterns, the modified EIR for the IRS communities was approximately 1.8 ib/p/yr, indicating a 69.9% reduction compared to the directly measured EIR. On the other hand, the control communities recorded a 16.5% decrease in EIR when adjusting the directly measured EIR from 16.2 ib/p/yr to 13.6 ib/p/yr (Table 4).

**Table 4:** Sporozoite rates and entomologic inoculation rates of *Anopheles gambiae* sensu lato collected by human landing catches in the control and indoor residual spraying intervention communities, June 2017–April 2019

Period of study	Number of mosquitoes analyzed	Sporozoite rate (%)	Directly measured^a^		HBO and parity adjusted^b^
			Mean monthly EIR	Annual^c^ EIR	Annual^c^ EIR
*IRS communities*					
Year 1	1209	0.17	0.32	3.19	1.64
Year 2	1133	0.35	0.91	9.08	2.06
Mean		0.26	0.61	6.14	1.85
*Control communities*					
Year 1	2574	0.47	1.37	13.66	13.34
Year 2	2151	0.51	1.88	18.86	13.80
Mean		0.49	1.62	16.26	13.57

*EIR* Entomological inoculation rate,* HBO* human behavior observation

^a^Directly measured EIR is estimated with directly measured biting rates from human landing catches

^b^HBO- and parity-adjusted EIRs refers to EIR estimation with HBO- and parity-adjusted biting rates

^c^Annual EIR = sum of monthly EIRs

## Discussion

Considering that the risk of mosquito bites is influenced by availability of host or other human factors [35], it is necessary to consider human behavior when estimating the risk of exposure in order to accurately quantify the impact of malaria vector control interventions. An understanding of both vector bionomics and corresponding human behavior [13, 36] is critical to determining the risk of vector-borne disease transmission and selecting the most appropriate interventions and evaluating their impact. It has long been established that malaria transmission is highly sensitive to adult mosquito survival or age, biting risk and vector infectivity [37, 38]. In this study, we estimated malaria transmission risk based on vector–human interactions in northern Ghana where IRS and ITNs have been deployed since 2008.

*Anopheles gambiae* was the predominant vector collected in this 2-year study and it was found to exist in sympatry with *An. coluzzii* and *An. arabiensis*. A notable decline in vector biting rates (HBRs) was observed in the control communities in the second year of the study. This decline in HBR could in part be attributed to the impact of a mass ITN distribution campaign organized by the National Malaria Control Program just before the peak malaria season in June 2018. Whereas the mass ITN campaign excluded all IRS intervention communities, all households in the comparison control (non-IRS) communities were covered. ITN coverage in the IRS communities was, however, maintained through the continuous distribution in health facilities. The observed decline in HBRs in the control communities following the ITN distribution in 2018 is consistent with the findings of Bayoh et al. [39] who found a dramatic decline in *An. gambiae* s.s. populations following increased coverage of insecticide-treated bed nets in Kenya [39]. In contrast, there was about 23% increase in the biting rates of *An. gambiae* s.l. in the IRS communities in year 2 when compared to year 1, with a significant spike in mosquito biting density in September 2018. The notable spike in mosquito biting density in the IRS communities coincided with flooding in IRS communities (under the Kumbungu district) and neighboring Savelugu, Mamprugu/Moagduri and West Mamprusi districts. The flooding, which occurred between late August 2018 and early September 2018, was caused by the spillage of the Bagre Dam in Burkina Faso [40, 41]. Floods and excessive rainfall are known to reduce mosquito populations by flushing out breeding sites [42, 43]. However, receding floodwaters and pooled water caused by heavy rainfall have also been found to contribute to abundance of temporary mosquito breeding habitats and subsequently to increased mosquito biting density 2–4 weeks after flooding or heavy rains [43–45]. The HLCs were conducted 3 weeks after the floods, suggesting that the receding floodwaters may have contributed to an increase in larval habitats and, consequently, a significant rise in mosquito biting densities in the IRS communities during year 2 of the study.

The general increase in EIR estimated in year 2 could be as a result of the increased vector biting rates observed in year 2. A similar observation was reported by Dia et al. in Dakar (Senegal) where malaria transmission has been sustained in certain suburbs of Dakar by recurrent flooding since 2005 [46]. These authors suggest that recurrent floods in the area permit a year-round persistence of *Anopheles arabiensis* larval habitats which maintain vector populations, thus increasing malaria transmission risk [46].

Historically, *An. gambiae* has been found to be primarily endophagic and endophilic [47]. However, data suggest that prolonged implementation of IRS and/or use of ITNs may induce exophagy [48–50], which undermines the efficacy of indoor-targeted interventions. Our findings suggest that in the study area in northern Ghana, *An. gambiae* tended to feed both indoors and outdoors when hosts were available. Overall, outdoor vector biting rates were slightly higher (though non-significant) in the IRS + ITN communities compared to the control (ITNs only) communities. This outdoor feeding tendencies of *An. gambiae* s.l. observed in IRS communities could be influenced by irritant effects of pirimiphos-methyl (in the insecticide Actellic 300CS) that was sprayed during the study period. This observation is consistent with findings in Côte d’Ivoire where pirimiphos-methyl deterred vectors from entering sprayed huts despite the commonly held belief that pirimiphos-methyl is considered to be a non-irritant insecticide [51]. It also supports the assertion that prolonged implementation of IRS and/or use of ITNs could induce exophagy, as previously reported in other studies [48–50],

Though the overall outdoor biting rates of the vector are not as significant as reported in other studies [11, 32, 52–54], the direct observation results indicate significant outdoor human night-time activities (outdoor sleeping and entertainments events) that could expose individuals to infective bites before bed time. However, individual risk can vary substantially depending on seasonality and human behavior. Our results suggest that outdoor sleeping during the dry season may not pose a high risk of malaria transmission due to very little mosquito biting activity. In contrast, during the rainy season, a significant number of people are exposed to mosquito bites outdoors during the evening hours, representing significant risk when mosquito sporozoite infection rates are high. When the biting rates for the IRS communities are adjusted for HBO and parity, only 3% of the total human–vector contact with potentially infective mosquitoes occurs indoors before bed time, while about 41% of the contact is outside houses. The human–vector contact before bed time is 8% versus 31% for indoors and outdoors, respectively, for the control communities. Therefore, the proportion of transmission that occurs outdoors could contribute to residual transmission and will likely require additional vector control, such as larval source management, spatial repellents, attractive targeted sugar baits and/or personal protective measures, to further reduce the burden of malaria in the study area as these tools target different behaviors. Additionally, the low ITN utilization, despite high ownership rates in the study area, suggests a need for intensified communication on social and behavior change to promote increased uptake and proper use of ITNs at the household level.

Despite the increased biting density in year 2 and the plastic feeding behavior of the main vector species in the area, our results show that IRS implementation and ITN usage led to a decrease in human exposure to potentially infective mosquito bites in the IRS communities, as opposed to the control communities. This decrease was observed even with only moderate ITN usage in the IRS communities. Given the long extrinsic incubation period of *Plasmodium* parasites inside *Anopheles* vectors, mosquito survivorship largely determines the number of secondary infections that could result from the primary source [37]; there is an increased risk of malaria transmission with increasing proportion of older mosquitoes within a given population [55]. Consequently, small reductions in longevity and biting rates of vectors could result in significant declines in vectorial capacity and epidemiological outcomes [31, 37]. This was confirmed by our study findings. Using parity as an indirect measure of mosquito age, a significantly younger mosquito population was observed in IRS communities as compared to control communities. Consequently, both directly estimated EIRs and HBO- and parity-adjusted EIRs remained comparatively lower in the IRS communities. These estimations could be as a result of the mosquito population in the IRS communities being predominated by a high proportion (52%) of younger (nulliparous) mosquitoes compared to the control communities where the mosquito population was much older. These findings conform with those from other studies across Africa which have shown that IRS significantly reduces vector longevity [56–58]. In contrast, the relatively higher usage of pyrethroid-only ITNs in the control communities could not significantly affect vector survival as well as EIRs and may not be an adequate measure to affect the capacity of vectors to transmit malaria [37]. It is possible that the highly endophilic vectors found in the control communities might have survived exposure to the pyrethroid-only ITNs due to their high pyrethroid resistance intensity, as reported in the predominant vector species in northern Ghana [59, 60]. Susceptibility tests conducted on adult female *An. gambiae* s.l. mosquitoes at multiple sites across the IRS and control study area showed resistance up to 10× the concentrations of alphacypermethrin and deltamethrin [22]. Results from follow-on synergist bioassays suggest that mono-oxygenases may be involved in the resistance mechanism. However, the vectors in the IRS communities remained susceptible to the pirimiphos methyl spray applied throughout the study period [22, 23, 61].

At the current ITN utilization rates of 38% and 57% in the IRS intervention and control communities, respectively, the proportion of all vector bites prevented by using an ITN is 33% and 49% for the IRS and control communities, respectively. Considering that most of human bites (51% in IRS vs. 40% in control communities) occurred indoors while people were asleep, hypothetically increasing ITN utilization to 100% would significantly increase personal protection and prevent up to 85% of potentially infective bites. However important barriers, such as heat and discomfort under the ITN, room shape and size [62, 63], remain important practical factors that hinder such large-scale utilization and consistent use of ITNs. Additionally, the inability of the pyrethroid-only nets to affect vector survival, the plastic feeding habits of the predominant vectors (i.e. feeding indoors/outdoors depending on host availability) and the considerable outdoor human activity during night-time imply that some transmission may occur beyond the scope of existing interventions. In a study that looked at the effect of pyrethroid resistance on ITN efficacy in Africa, Churcher et al. found that adding a synergist (piperonyl butoxide [PBO]) to the regular ITNs could avert up to 0.5 clinical cases per person per year in places, with better epidemiological outcomes [64]. Therefore, the use of new-generation ITNs, such as those containing PBO or dual active ingredient mixtures in addition to intensified social and behavior change communication, could result in greater epidemiological impact compared to pyrethroid-only ITNs.

The findings from this study confirm that vector bionomics alone do not reveal true transmission exposure risk. HBO and parity adjustments suggest that without accounting for human behavioral patterns and the effect of interventions on vector survival we could be overestimating malaria transmission risk (EIRs) by about 70% in the IRS communities and about 17% in ITN only-control sites. Such high EIRs estimated with the directly measured HBRs may suggest that interventions might not be working and undermine the impact of existing interventions. By adjusting the HBR for human behavior and parity and using these to estimate EIRs, we determined a more accurate estimate of the effect of vector control interventions on malaria vectors and risk of transmission. The findings of the study suggest that the combination of IRS and ITNs have a greater impact on malaria transmission risk than ITNs alone. They also reveal that additional interventions would be needed to tackle possible transmission that occurs beyond the reach of IRS and ITNs, due to vector feeding plasticity and human behavior patterns. By considering this information, we can ultimately tailor vector control interventions to meet specific needs and deliver appropriate messaging to communities through innovative and effective communication strategy.

## Conclusions

The study has shown that IRS continues to have a significant impact on vector longevity and malaria transmission, as determined by entomological indicators, despite the varying degree of outdoor biting encountered in the IRS communities. By measuring multiple variables that define the dynamics of malaria transmission, namely net ownership and use, human sleeping behavior, seasonality, mosquito biting rate, infectivity and longevity, we accurately quantified exposure risk and the true impact of current vector control interventions in the study area. The results of the study indicate that ITNs are more effective when combined with IRS than when deployed alone in northern Ghana. However, we identified protection gaps due to human and vector behavior patterns that allow significant human–vector interaction beyond the current control interventions. Therefore, it is essential to consider human behavior when selecting, implementing and evaluating the effectiveness of malaria control interventions. Additionally, reinforcing effective communication on the need for behavioral change is crucial to maximize the uptake and efficacy of vector control interventions.

## Supplementary Information

**Additional file 1. Table S1**: List of study sites and their IRS history from 2008 to 2020. **Table S2**: Total number of *Anopheles* mosquitoes collected during the study period. **Table S3**: Proportion of parous females of *An. gambiae* s.l. by HLCs, June 17–April 2019. **Figure S1**: Estimates of monthly parity rates of *An. gambiae* s.l. by for IRS intervention and control communities, June 2017 to April 2019. **Figure S2**: Mean monthly indoor and Outdoor human biting rates of *An. gambiae* s.l. for IRS intervention and control sites, June 2017 to April 2019. **Figure S3**: Mean hourly bites per person and hourly sporozoite rates for *An. gambiae* s.l. in **a** IRS and **b** control sites in the rainy seasons of year 1 and 2.

**Additional file 2**.** Form S1**: Household direct observation data collection form.

## Data Availability

The datasets used in this study are available from the corresponding author on reasonable request.

## Abbreviations

EIR: Entomological inoculation rate
HBO: Human behavior observations
HBR: Human biting rate
HLCs: Human landing catches
IRS: Indoor residual spraying
ITN: Insecticide-treated nets
b/p/n: Bites per person per night
