# Supplementary material for: Estimating malaria transmission risk through surveillance of human–vector interactions in northern Ghana
Source: Parasit Vectors. 2023 Jun 19;16:205. doi: 10.1186/s13071-023-05793-2 (PMC10280856; doi:10.1186/s13071-023-05793-2)
Supplement: Supplementary file 1 — Additional file 1. Table S1: List of study sites and their IRS history from 2008 to 2020. Table S2: Total number of Anopheles mosquitoes collected during the study period. Table S3: Proportion of parous females of An. gambiae s.l. by HLCs, June 17–April 2019. Figure S1: Estimates of monthly parity rates of An. gambiae s.l. by for IRS intervention and control communities, June 2017 to April 2019. Figure S2: Mean monthly indoor and Outdoor human biting rates of An. gambiae s.l. for IRS intervention and control sites, June 2017 to April 2019. Figure S3: Mean hourly bites per person and hourly sporozoite rates for An. gambiae s.l. in a IRS and b control sites in the rainy seasons of year 1 and 2. [file 13071_2023_5793_MOESM1_ESM.docx]

Supplementary 1:

**Estimating Malaria Transmission Risk through Surveillance of Human-Vector Interactions in Northern Ghana**

**Sylvester Coleman** ^1,4,8^ *****, Yemane Yihdego^1^, Frank Gyamfi^1^, Lena Kolyada^1^, Jon Eric Tongren^2^, Sixte Zigirumugabe^3^, Dominic B. Dery^3^, Kingsley Badu^4^, Kwasi Obiri-Danso^4^, Daniel Boakye^5^, Daniel Szumlas^6^, Jennifer S. Armistead^7^, Samuel K. Dadzie^5^

^1^U.S. President’s Malaria Initiative VectorLink Project, Accra, Ghana; ^2^U.S. President’s Malaria Initiative, Malaria Branch, U.S. Centers for Disease Control and Prevention, Accra, Ghana; ^3^U.S. President’s Malaria Initiative, U.S. Agency for International Development, Accra, Ghana; ^4^Kwame Nkrumah University of Science and Technology, Kumasi, Ghana.

^5^Noguchi Memorial Institute for Medical Research, University of Ghana, Legon, Accra, Ghana; ^6^Armed Forces Pest Management Board 172 Forney Road, Forest Glen Annex, Silver Spring, MD 20910; ^7^U.S. President’s Malaria Initiative, U.S. Agency for International Development, Washington D.C., United States; ^8^Department of Vector Biology, Liverpool School of Tropical Medicine, Pembroke Place, Liverpool, L3 5QA, UK.

Table S1: List of sites and their IRS history from 2008 to 2020

| Districts | Site | INSECTICIDE SPRAYED AND SPRAY COVERAGE | | | | | | | | | | |
| --- | --- | --- | --- | --- | --- | --- | --- | --- | --- | --- | --- | --- |
|  |  | 2008 | 2009 | 2010 | 2011 | 2012 | 2013 | 2014 | 2015 | 2016 | 2017 | 2018 |
| Kumbungu District (KUD) | Gbullung | ACy | ACy | DM | ACy | ACy | *NSp* | *NSp* | PM | PM | PM | PM |
|  |  | (91%) | (93%) | (96%) | (91%) | (93%) | *NSp* | *NSp* | (94%) | (93%) | (89%) | (90%) |
|  | Gupanarigu | ACy | ACy | DM | ACy | ACy | *NSp* | *NSp* | PM | PM | PM | PM |
|  |  | (93%) | (95%) | (95%) | (93%) | (95%) | *NSp* | *NSp* | (93%) | (96%) | (94%) | (95%) |
| Tamale Metropolis (TML) | Kulaa | *NSp* | | | | | | | | | | |
|  | Tugu | *NSp* | | | | | | | | | | |
| *Note:* ACy=alpha-cypermethrin; DM=deltamethrin; PM=pirimiphos methyl NSp=not sprayed.  *Spray coverage*: the proportion of the total number of actual structures sprayed or population protected out of the total number of structures or population targeted. | | | | | | | | | | | | |

| **Species** | **Type of Treatment** | **Sentinel site** | **Indoor landing catch** | |  |  | **Outdoor landing catch** | | | | | | | **Grand Total** | |  |
| --- | --- | --- | --- | --- | --- | --- | --- | --- | --- | --- | --- | --- | --- | --- | --- | --- |
|  |  |  |  | **Year 1** | **Year 2** |  | **Year 1** |  | | **Year 2** | |  | |  | |  |
|  |  |  | **Dry** | **Rainy** | **Dry** | **Rainy** | **Dry** | **Rainy** | | **Dry** | | **Rainy** | |  | |  |
| **No of HLC nights** | | | 20 | 20 | 20 | 20 | 20 | 20 | | 20 | | 20 | |  | |  |
| **No of Man-nights** | | | 200 | 200 | 200 | 200 | 200 | 200 | | 200 | | 200 | |  | |  |
| *An. gambiae* s.l. | IRS | Gbullung | 3 | 1,615 | 18 | 1,692 | 15 | 2,140 | | 17 | | 2,338 | | 7,838 | |  |
|  |  | Gupanarigu | 2 | 2,312 | 11 | 2,807 | 1 | 1,757 | | 10 | | 2,762 | | 9,662 | |  |
|  | Control | Kulaa | 52 | 5,398 | 55 | 3,359 | 35 | 4,502 | | 53 | | 2,729 | | 16,183 | |  |
|  |  | Tugu | 219 | 3,675 | 65 | 2,175 | 231 | 3,648 | | 41 | | 1,869 | | 11,923 | |  |
|  | Sub-total | | **276** | **13,000** | **149** | **10,033** | **282** | | **12,047** | | **121** | | **9,698** | | **45,606** | |
| *An. funestus* s.l. | IRS | Gbullung | 0 | 25 | 0 | 20 | 0 | 56 | | 4 | | 19 | | 124 | |  |
|  |  | Gupanarigu | 0 | 30 | 0 | 10 | 0 | 31 | | 0 | | 9 | | 80 | |  |
|  | Control | Kulaa | 0 | 2 | 1 | 0 | 0 | 4 | | 0 | | 1 | | 8 | |  |
|  |  | Tugu | 0 | 2 | 0 | 0 | 0 | 0 | | 0 | | 0 | | 2 | |  |
|  | Sub-total | | **0** | **59** | **1** | **30** | **0** | | **91** | | **4** | | **29** | | **214** | |
| *An. nili* | IRS | Gbullung | 0 | 32 | 0 | 47 | 0 | 56 | | 0 | | 97 | | 232 | |  |
|  |  | Gupanarigu | 0 | 0 | 0 | 11 | 0 | 2 | | 0 | | 21 | | 34 | |  |
|  | Control | Kulaa | 0 | 108 | 0 | 630 | 0 | 114 | | 0 | | 708 | | 1560 | |  |
|  |  | Tugu | 0 | 83 | 0 | 284 | 0 | 80 | | 0 | | 362 | | 809 | |  |
|  | Sub-total | | **0** | **223** | **0** | **972** | **0** | | **252** | | **0** | | **1,188** | | **2,635** | |
| *An. pharoensis* | IRS | Gbullung | 0 | 3 | 0 | 5 | 0 | 17 | | 0 | | 8 | | 33 | |  |
|  |  | Gupanarigu | 0 | 1 | 0 | 2 | 0 | 6 | | 0 | | 0 | | 9 | |  |
|  | Control | Kulaa | 0 | 19 | 1 | 4 | 0 | 26 | | 1 | | 6 | | 57 | |  |
|  |  | Tugu | 0 | 31 | 0 | 22 | 0 | 107 | | 0 | | 15 | | 175 | |  |
|  | Sub-total | | **0** | **54** | **1** | **33** | **0** | | **156** | | **1** | | **29** | | **274** | |
| **Grand Total** |  |  | **276** | **13,336** | **151** | **11,071** | **282** | **12,546** | | **126** | | **10,947** | | **48,735** | |  |

Table S2: Total number of *Anopheles* mosquitoes collected during the study period

Only 6 *An. rufipes* (3 from each site) were collected

Table S3: Proportion of parous females of *An. gambiae* s.l. by HLCs, June 17 – April 2019

| **Study Site and Period of Collection** | **Total Dissected** | **# Parous** | **Parity Rates** | | |
| --- | --- | --- | --- | --- | --- |
|  |  |  | **Mean** | **95% Confidence Interval** | |
|  |  |  |  | **Lower** | **Upper** |
| **IRS Intervention** | | | | | |
| Year 1 | 893 | 357 | 39.98% | 36.8% | 43.19% |
| Year 2 | 1441 | 685 | 47.54% | 45.0% | 50.11% |
| **Control** |  |  |  |  |  |
| Year 1 | 1866 | 1402 | 75.13% | 73.2% | 77.10% |
| Year 2 | 1360 | 911 | 66.99% | 64.5% | 69.48% |
|  |  |  |  |  |  |
| **Total** |  |  |  |  |  |
| IRS Intervention | 2334 | 1042 | 44.64%***^†^*** | 42.6% | 46.66% |
| Control | 3226 | 2313 | 71.70%***^†^*** | 70.1% | 73.25% |
| *Results for Z test of proportion for parity IRS vs Control:*  *Pooled sample proportion: 0.603417266, p-value: <0.001 Z test statistic: -20.35,*  *^†^Differences in mean parity significant at 0.05 sig. level* | | | | | |


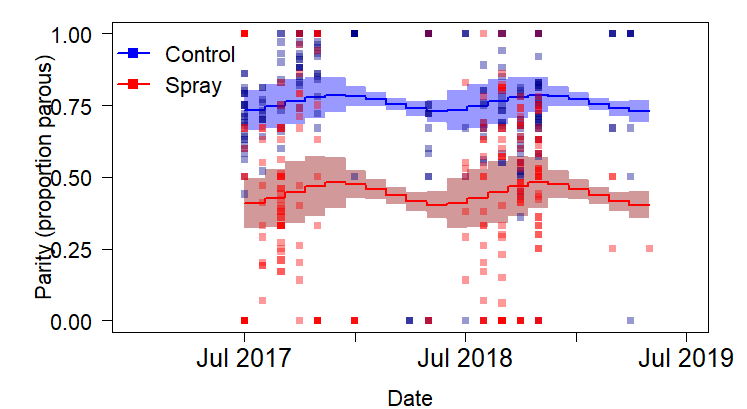


Figure S1: Estimates of monthly parity rates of *An. gambiae* s.l. for IRS intervention and control sites, June 2017 to April 2019.


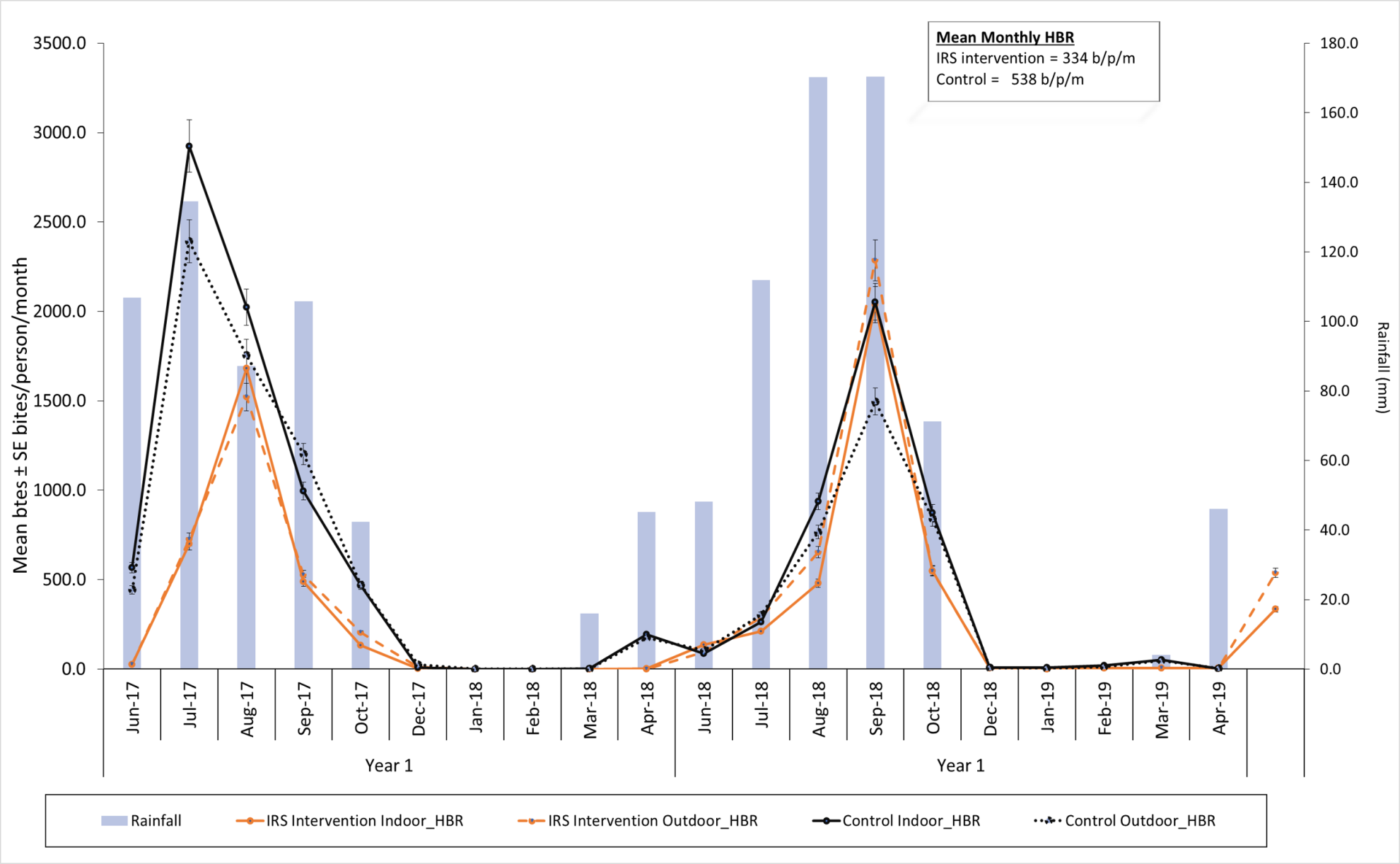
 Figure S2: Mean monthly indoor and outdoor human biting rates of *An. gambiae* s.l. for IRS intervention and control sites, June 2017 to April 2019. Mean bites per person per month (b/p/m) was 334 for the IRS intervention communities and 538 b/p/m for the control communities


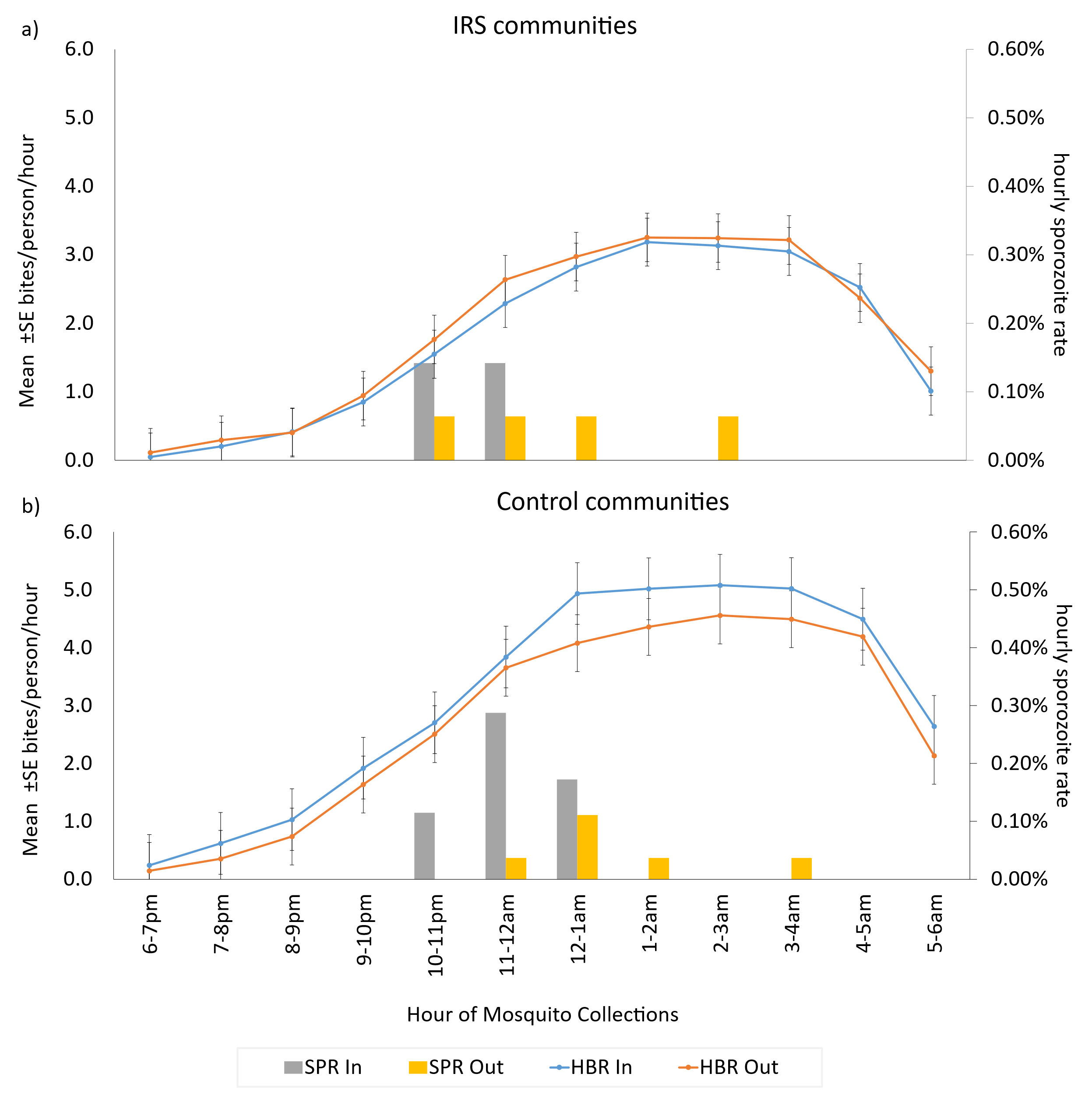


Figure S3: Mean hourly bites per person and hourly sporozoite rates for *An. gambiae* s.l. in a) IRS and b) control sites in the rainy seasons of year 1 and 2. Peak indoor (blue lines) and outdoor (orange lines) hourly HBRs were observed between 1:00 AM and 3:00 AM in both IRS and control sites. Peak indoor (grey bars) and outdoor (yellow bars) sporozoite rates (SPR) were observed between 10:00 PM and 4:00 AM.
