# Supplementary material for: Estimating malaria transmission risk through surveillance of human–vector interactions in northern Ghana
Source: Parasit Vectors. 2023 Jun 19;16:205. doi: 10.1186/s13071-023-05793-2 (PMC10280856; doi:10.1186/s13071-023-05793-2)
Supplement: Supplementary file 2 — Additional file 2. Form S1: Household direct observation data collection form. [file 13071_2023_5793_MOESM2_ESM.doc]

Supplementary 2:

**Estimating Malaria Transmission Risk through Surveillance of Human-Vector Interactions in Northern Ghana**

**Sylvester Coleman** ^1,4,8^ *****, Yemane Yihdego^1^, Frank Gyamfi^1^, Lena Kolyada^1^, Jon Eric Tongren^2^, Sixte Zigirumugabe^3^, Dominic B. Dery^3^, Kingsley Badu^4^, Kwasi Obiri-Danso^4^, Daniel Boakye^5^, Daniel Szumlas^6^, Jennifer S. Armistead^7^, Samuel K. Dadzie^5^

^1^U.S. President’s Malaria Initiative VectorLink Project, Accra, Ghana; ^2^U.S. President’s Malaria Initiative, Malaria Branch, U.S. Centers for Disease Control and Prevention, Accra, Ghana; ^3^U.S. President’s Malaria Initiative, U.S. Agency for International Development, Accra, Ghana; ^4^Kwame Nkrumah University of Science and Technology, Kumasi, Ghana.

^5^Noguchi Memorial Institute for Medical Research, University of Ghana, Legon, Accra, Ghana; ^6^Armed Forces Pest Management Board 172 Forney Road, Forest Glen Annex, Silver Spring, MD 20910; ^7^U.S. President’s Malaria Initiative, U.S. Agency for International Development, Washington D.C., United States; ^8^Department of Vector Biology, Liverpool School of Tropical Medicine, Pembroke Place, Liverpool, L3 5QA, UK.

**FORM S1 : HOUSEHOLD DIRECT OBSERVATION FORM**

**Direct Observation of Night-time Household Activity and Use of Bed Nets**

Time observation starts: _____________

Time observation ends: ______________

**NB**: *All the information recorded o this form should be information observed directly by the observer. (Do not include information that is only reported to the observer, unless the question indicates otherwise.)*

**List of Household Members**

| **Member and position/relationship to others in the household** (e.g., “wife of household head’s son,” “daughter of 1 & 2” or “friend of 1”) | **Gender & Age** | **If female, indicate if pregnant (y/n).** | **Indicate the time he/she remains indoor for the rest of the night** | **Net ownership and use Indoors:** | | **Slept outdoor? (y/n) If yes, Time?** | **Net ownership and use outdoors:** | |
| --- | --- | --- | --- | --- | --- | --- | --- | --- |
|  |  |  |  | **Own?** | **Use?** |  | **Own?** | **Use?** |
| 1. Head of household |  |  |  |  |  |  |  |  |
| 2. |  |  |  |  |  |  |  |  |
| 3. |  |  |  |  |  |  |  |  |
| 4. |  |  |  |  |  |  |  |  |
| 5. |  |  |  |  |  |  |  |  |
| 6. |  |  |  |  |  |  |  |  |
| 7. |  |  |  |  |  |  |  |  |
| 8. |  |  |  |  |  |  |  |  |
| 9. |  |  |  |  |  |  |  |  |

**10. Please list primary outdoor activities (Insert and number extra pages as necessary.) Continue listing activities until the end of the observation.**

*For example (alternatively, use the numbers from the household member list to identify people):*

*17:00 – 19:00 Head of household’s wife begins to cook in the kitchen. Daughter age 4 helps,*

*16:00 – 19:00 Boy age 6, plays outside*

*19:00- 20:30 Family eats. Part of time inside, most of time outside*

*21:00 – 24:00 Husband and second wife attend funeral at XXX outside. 20 people from other families attend*

**Reminder to record the following activities: indoor and outdoor sleeping, group social activities that occur outdoors at night, nocturnal occupational activities that occur outdoors, non-intimate domestic and recreational household activities, activities related to bed nets, and evidence of reactivity. interactions and personal and private actions (such as using toilets and bathing) should *not* be recorded.**

| Time | Activity |
| --- | --- |
|  |  |

***Before leaving, please ensure that all of the above questions/sections have been answer.***
